# Time Dependency of Chemodiversity and Biosynthetic Pathways: An LC-MS Metabolomic Study of Marine-Sourced *Penicillium*

**DOI:** 10.3390/md14050103

**Published:** 2016-05-21

**Authors:** Catherine Roullier, Samuel Bertrand, Elodie Blanchet, Mathilde Peigné, Thibaut Robiou du Pont, Yann Guitton, Yves François Pouchus, Olivier Grovel

**Affiliations:** 1 Faculty of Pharmacy, University of Nantes, EA 2160-Mer Molécules Santé, 9 rue Bias BP 53508, Nantes-cedex 1 44035, France; samuel.bertrand@univ-nantes.fr (S.B.); blanchet.elodie1@gmail.com (E.B.); mathildepeigne@hotmail.fr (M.P.); thibaut.robiou@univ-nantes.fr (T.R.d.P.); yann.guitton@oniris-nantes.fr (Y.G.); yves-francois.pouchus@univ-nantes.fr (Y.F.P.); olivier.grovel@univ-nantes.fr (O.G.); 2 ThalassOMICS, Plateforme Corsaire, Biogenouest, Nantes 44035, France; 3 Sorbonne Universités, UPMC Université Paris, USR 3579, LBBM, Observatoire Océanologique, Banyuls-sur-Mer 66650, France; 4 Laboratoire d’Etude des Résidus et Contaminants dans les Aliments (LABERCA), LUNAM Université, Oniris, Nantes 44307, France

**Keywords:** biosynthesis, griseofulvin, kinetics, LC-MS, marine fungi, natural products, time-scale metabolomics, untargeted metabolomics

## Abstract

This work aimed at studying metabolome variations of marine fungal strains along their growth to highlight the importance of the parameter “time” for new natural products discovery. An untargeted time-scale metabolomic study has been performed on two different marine-derived *Penicillium* strains. They were cultivated for 18 days and their crude extracts were analyzed by HPLC-DAD-HRMS (High Performance Liquid Chromatography-Diode Array Detector-High Resolution Mass Spectrometry) each day. With the example of griseofulvin biosynthesis, a pathway shared by both strains, this work provides a new approach to study biosynthetic pathway regulations, which could be applied to other metabolites and more particularly new ones. Moreover, the results of this study emphasize the interest of such an approach for the discovery of new chemical entities. In particular, at every harvesting time, previously undetected features were observed in the LC-MS (Liquid Chromatography-Mass Spectrometry) data. Therefore, harvesting times for metabolite extraction should be performed at different time points to access the hidden metabolome.

## 1. Introduction

It is generally admitted that only a small part of the marine fungal metabolic potential is observed under classical experiments consisting in cultivating one strain in axenic conditions on a common medium over a definite period of time. This greatly limits the potential of drug discovery from fungi [1]. To overcome this issue, many research teams have then investigated ways to unravel cryptic biosynthetic pathways to access a wider chemodiversity [2,3,4]. Thus far, different methods to awake silent biosynthetic gene clusters have been performed such as targeted mutagenesis [5,6,7,8], changes in culture media [9,10,11,12], the use of different types of elicitors and epigenetic modifiers [13,14], as well as co-cultivation of organisms [15,16,17,18]. The chemical profiling of every extract generated by such approaches usually generates large amounts of data, which need powerful tools to be analyzed and highlight the newly disclosed metabolome. This corresponds to the advent of “Metabolomics” studies, for which bioinformatics tools have proven to be essential for data analysis. This field of research combining natural product chemistry and metabolomics studies is clearly growing up [19,20,21,22,23].

Following the above-mentioned approaches for secondary metabolites induction, metabolomic studies are based on untargeted metabolite profiling of fungal extracts replicates using mainly direct ionization-mass spectrometry (DI-MS), gas and liquid chromatography coupled to mass spectrometry (GC-MS and LC-MS) or nuclear magnetic resonance (NMR) spectroscopic analyses [24]. These analytical methods, using generic approaches, aim to be as comprehensive as possible to be able to detect any changes in the chemical composition of the fungal extract in relation to the applied induction strategies after a selected period of time.

Metabolism is a function of the development state of an organism and consequently changes over time. For instance, in every organism, biochemical processes vary along the daytime. Most of them adapt their metabolism to the different environmental fluctuations of the diurnal rhythm [25]. Furthermore, most biosynthetic processes, which are enzyme-catalyzed reactions, are time-dependent because the enzymes involved have to be synthesized, modified or degraded. Consequently, many metabolites have a finite half-life, and their production and presence will naturally vary dynamically through time. The metabolome expressed by fungi in cultures may then vary depending on incubation time after inoculation but, so far, few studies have compared metabolic profiles through time. Only few hints of this metabolic variation in fungi have been described in recent studies. For instance, when growing *Cordyceps militaris* on germinated soybeans, Choi *et al*. have shown differences in metabolite composition over time, which had allowed the identification of novel compounds [26]. In the same way, when co-cultivating strains belonging to *Fusarium* and *Aspergillus* genera, Bertrand *et al*. have highlighted two features that were detected after four to seven days of culture and disappeared after nine days [27]. These studies confirmed the dynamic nature of the fungal metabolism and the potential novelty hidden in the time frame. Investigation of the parameter “time” in time-course metabolomic studies to unravel hidden metabolome would then be a new approach to access a wider chemodiversity.

In complement to proteomics and transcriptomics, time-series metabolomic studies appear to be an interesting way to investigate metabolism and biosynthetic pathways [28,29]. However, as time-course measurements are interconnected by nature, they are very complex to analyze and multivariate modeling of dynamic metabolomics data remains challenging as discussed in the literature [30,31,32,33]. Most examples given in the literature correspond to targeted analyses which purpose is to follow the behavior of an organism after a treatment or a stress [34,35,36,37,38]. To our knowledge, no untargeted investigation of metabolic profiles through time, particularly focused on marine fungi has been performed so far.

In the present work, an 18-day time-scale metabolomic study has been performed on two different marine-derived *Penicillium* strains, grown on agar plates. The crude extracts, obtained each day, were analyzed by high performance liquid chromatography coupled to a diode array detector and a high-resolution mass spectrometer (HPLC-DAD-HRMS). The resulting data were analyzed through metabolomic approaches to highlight differences in terms of intermediate and final metabolites, as well as their production behavior through time. As both strains produced griseofulvin, an antifungal drug that is still on the market and regains interest in the field of cancer [39,40], its biosynthetic pathway was further explored with a particular focus on its time dependency. A more general discussion about the chemodiversity of fungal metabolites and the assessment of their novelty through time is here given, which is of particular interest for natural products chemists.

## 2. Results and Discussion

**Figure 1:**
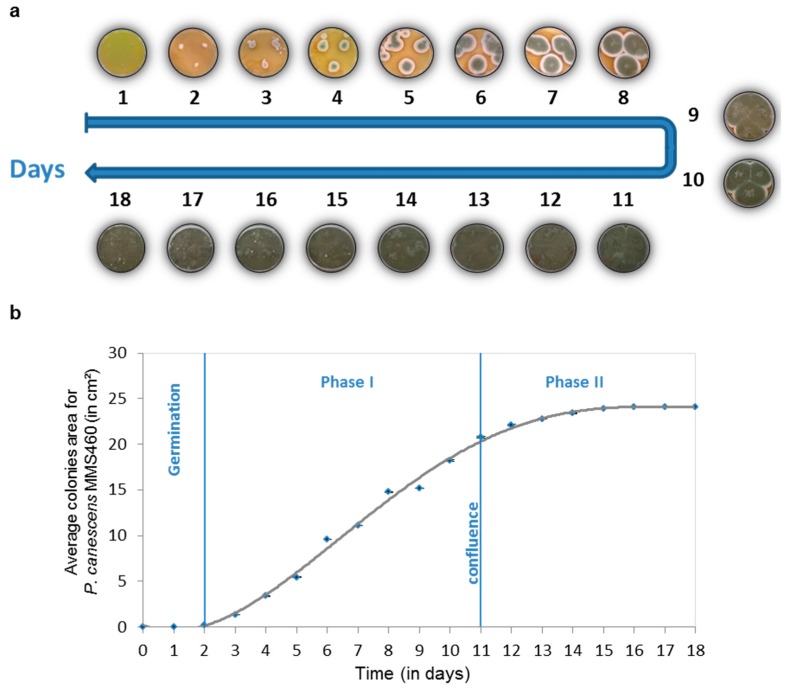
Morphological aspect of *Penicillium canescens* MMS460 cultures from Day 1 to Day 18 (**a**); and its associated fungal growth curve (**b**).

During fungal growth on agar, obvious morphological aspect changes occur. The mycelium spreads in all directions from the seeding point, due to a highly polarized process of hyphal tip extension, and forms typical circular colonies, which diameter can be followed through time to assess the fungal growth (as shown for *Penicillium canescens* MMS460 in Figure 1) [41,42,43]. During the first two days, which correspond to germination no obvious growth could be measured. From the third day, colonies began to expand exponentially (Phase I), with the central part forming dark-green spores. Around Day 11, the mycelium covered the whole plate with colonies attaining confluence and the growth curve reaching a plateau (Phase II). Macroscopic modifications of the fungal colonies in terms of size and color are obviously the result of molecular changes and differences in metabolites production. To assess these variations at the molecular level, extracts of two different strains on an 18-day period were analyzed. In this series of experiments, a three-plugs micro-extraction method was employed [44]. This allowed obtaining crude extracts in a sufficient amount for LC-MS profiling with good representation of what the composition of a global extract (whole medium and mycelium) would be. Metabolite production profiles over time were then first analyzed in an untargeted approach to highlight variations during the fungal growth. In a second part, a targeted analysis of biosynthetic intermediates of griseofulvin was performed to emphasize the interest of such an approach in the general understanding of biosynthetic pathways. 

### 2.1. Time Dependence of Metabolic Profiles

#### 2.1.1. General Observations

**Figure 2:**
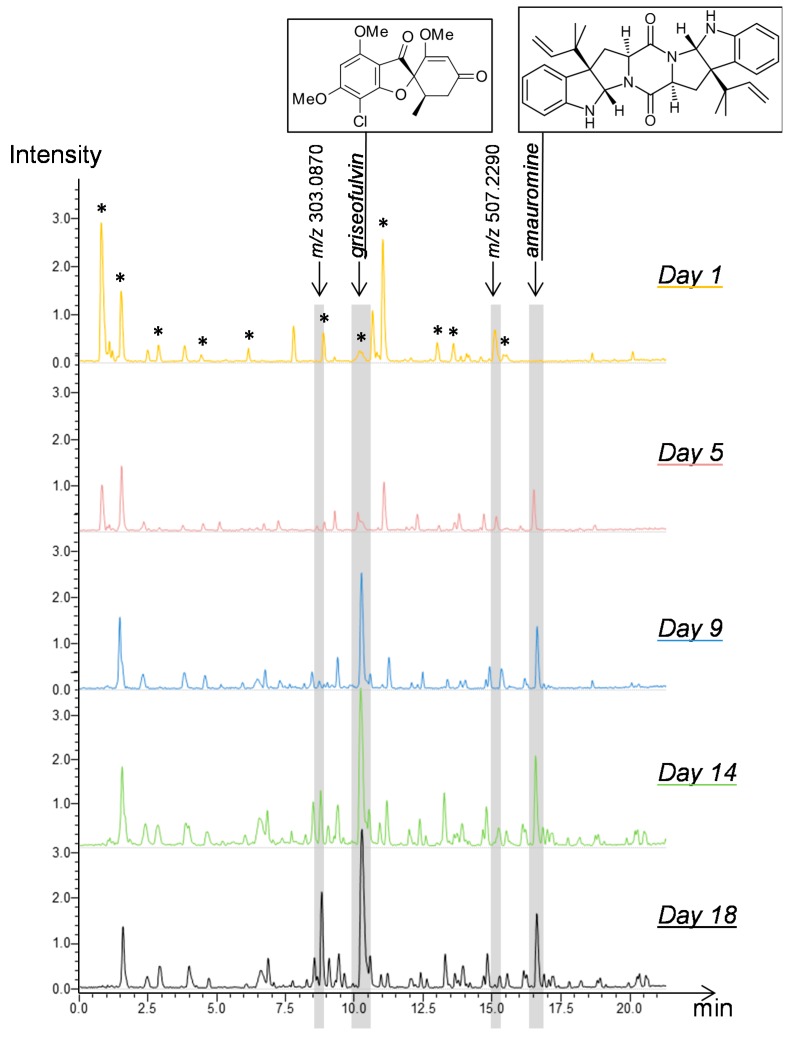
LC-(+)ESI-HRMS (Liquid chromatography-electron spray ionization in positive mode-high resolution mass spectrometry) traces (base peak chromatograms) obtained for crude extracts at selected time points over the 18 consecutive day period, from the culture of marine-derived *Penicillium canescens* MMS460. *m/z* 353.078 at 10.5 min and *m/z* 509.2907 at 16.8 min were identified as griseofulvin and amauromine, respectively; *m/z* 303.0870 at 9.1 min was annotated desmethyl-dehydro-dechloro-griseofulvin; and *m/z* 507.229 at 15.4 min could not be annotated. Peaks from the culture medium are highlighted (*).

The metabolic profile of each extract was obtained by high performance liquid chromatography coupled to high-resolution mass spectrometry (HPLC-HRMS) analysis. In the case of *P. canescens*, the direct observation of the obtained chromatograms highlighted some differences (Figure 2). For example, the peak corresponding to *m/z* 507.229 at 15.4 min was present at Day 1 and diminished from Day 5. Contrarily, some peaks appeared later, from Day 5 for *m/z* 509.2907 at 16.8 min (amauromine), from Day 9 for *m/z* 353.078 at 10.5 min (griseofulvin) and from Day 14 for *m/z* 303.0870 at 9.1 min. It is interesting to note that even at Day 1, while no growth was visually observed (or measurable), compounds not originating from the culture medium were already detected within the extracts (such as *m/z* 507.2290 at 15.4 min). This may correspond to initial seeding composition or early metabolic activity, but both cases are difficult to differentiate based on acquired data. Peaks from the culture medium then decrease in the following days, showing a dilution effect among other metabolites produced by the fungus and probable consumption or degradation. Moreover, more peaks could be observed in the latter profiles in comparison to those acquired at earlier time-points. This indicated that the richness of the samples was increasing through time. Therefore, a deeper computer-aided analysis of the chromatograms was performed to show additional time-based modifications of metabolite composition.

#### 2.1.2. In-Depth Analysis

**Figure 3:**
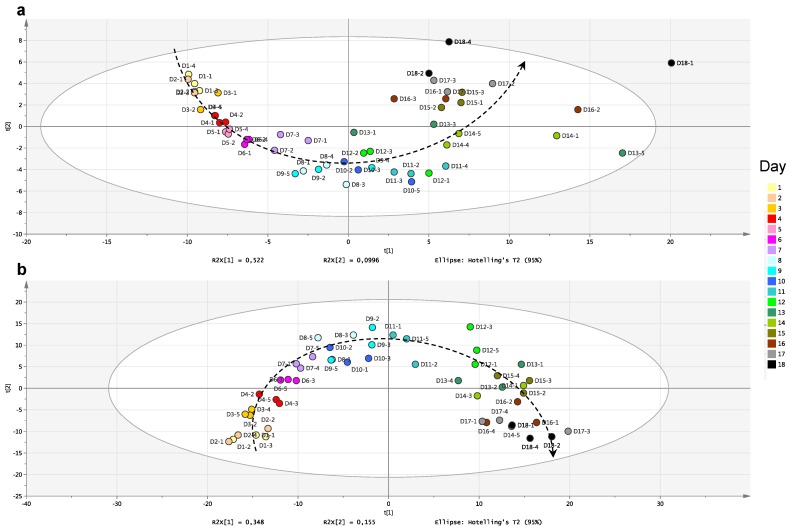
Principal component analysis (PCA) obtained from LC-(+)ESI-HRMS profiles corresponding to the extracts of marine-derived fungi from Day 1 (D1) to Day 18 (D18), (3 replicates per day) based on features (*m/z* at Rt associated to its normalized peak area) detected in LC-MS chromatograms: (**a**) *P. canescens* MMS460; and (**b**) *Penicillium* sp. MMS388.

**Figure 4:**
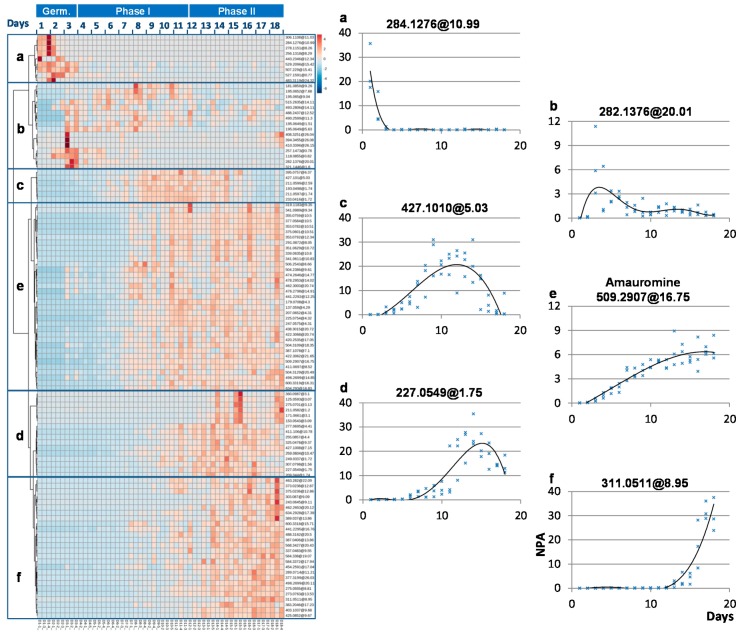
Hierarchical clustering of the features observed from Day 1 (D1) to Day 18 (D18) in a marine-derived *P. canescens* MMS460 in the positive mode (**left**), with scatterplots of selected representative features named by their corresponding *m/z* and retention time (*m/z* at Rt) with their fitted trend curves (x-axis representing harvesting days and y-axis representing normalized peak area (NPA) in the extracts) for the six highlighted categories of compound behaviors (**right**): (**a**) early compounds present from the spore germination and decreasing rapidly; the compounds with a typical Gaussian profile with a maximum concentration (**b**) at the beginning of phase I; (**c**) around confluence; or (**d**) during phase II of the fungus growth; (**e**) the compounds delayed in apparition with a tendency to accumulate; and (**f**) the late compounds clearly arising at a very late stage during phase II.

To better assess the chemodiversity produced by the two studied fungi, their profiles were analyzed using a comprehensive untargeted metabolomic approach, focusing on the “time” parameter. All the features detected by LC-MS (*m/z* at Rt associated to its peak area) were extracted by an automatic peak detection procedure [45]. Then the resulting data matrix was explored focusing on the detection of the changes in the crude extracts over time. The principal component analyses (PCA) constructed on these data showed a drift from Day 1 to Day 18 following a trajectory that runs from left to right of the figures in a semicircle shape (Figure 3). This indicated the ordinal structural characteristics in the data, as reported for other kinetic studies [29]. This highlighted the sequential apparition and disappearance of compounds in the extract. This drift through the first axis represented about 35%–50% of the explained variances showing the importance of this time-dependent evolution. This was further emphasized when the dispersion of data was presented as a hierarchical clustering analysis; this approach particularly highlighting the features presenting similar time behavior (Figure 4). This resulted in revealing six different characteristic behaviors corresponding to: (a) early compounds present from the spore germination and decreasing rapidly; the compounds with a typical Gaussian profile with a maximum concentration (b) at the beginning of phase I; (c) around confluence; or (d) during phase II of the fungus growth; (e) the compounds delayed in apparition with a tendency to accumulate; and (f) the late compounds clearly arising at a very late stage during phase II.

Some of the highlighted categories of compound behaviors over time could be related to classically reported kinetics of production in the literature, such as for scopularide B in the mutant *Microascus brevicaulis* LF580-M26 [46] and citrinin in *Monascus ruber* [47] (category **c**), for cyclosporine A and C in *Tolypocladium inflatum* [48] (category **d**), or for lovastatin in *Aspergillus terreus* [49] (category **e**). In this study, some identified compounds such as amauromine (*m/z* 509.2907 at 16.75 min) or griseofulvin (*m/z* 353.0782 at 10.5 min) fell in these classical categories, (**e**) and (**d**), respectively. Moreover, this study highlighted some unclassical kinetic production profiles, which clearly pointed out the differences between young (1–6 days) and old cultures (14–18 days) for both strains (for hierarchical clustering of features from MMS388, see Supplementary Materials). As revealed by Figure 4, this corresponds to metabolites present at a very early stage—behavior (a) and (b)—which are on most cases not detected anymore in later stages, or to metabolites arising very late in the experiment—behavior (f)—such as the desmethyl-dehydro-dechloro-griseofulvin (*m/z* 303.0870 at 9.1 min) or the non-annotated feature *m/z* 311.0511 at 8.95 min. It is interesting to note that most of these specific features from categories (**a**), (**b**) and (**f**), could not be identified, potentially meaning that because they do not occur at usual harvesting times, they may have never been isolated.

Additionally, in the case of behavior (c), compounds are not detected at the early stage of the culture, neither in the later stage. This particularly highlights the time dependence of metabolites production in fungi as already revealed in *Cordyceps militaris* [26]. In more complex experimental designs corresponding to fungal co-culture, the specific induction of compounds in relation to interspecies cross-talk could also be very time specific [27]. These results may explain the apparent difficulties observed by natural product chemists when working with fungi, reported as a “capricious behavior” by Williams *et al*. [50]. Therefore, the time dependence of metabolite production is a key parameter when exploring the whole chemical diversity produced by one organism. This evolution is very likely related to the succession of biochemical reactions representing the natural products biosynthetic pathways.

### 2.2. Case Study of Griseofulvin Biosynthesis

**Figure 5:**
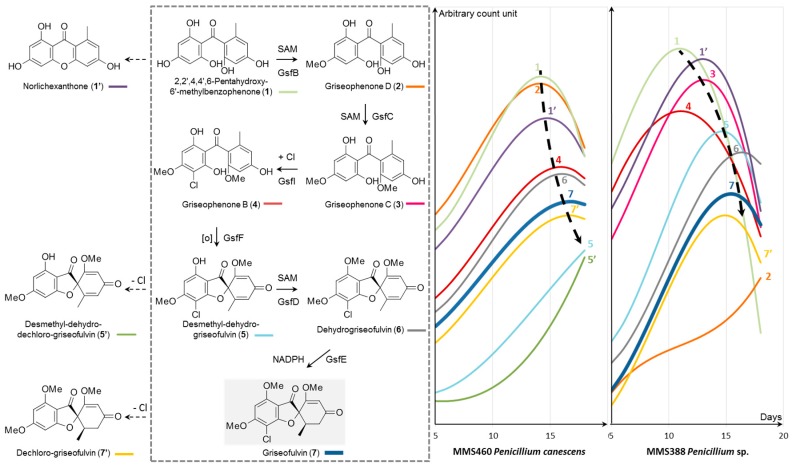
Griseofulvin biosynthesis (adapted from Cacho *et al*. [51]) study by superimposition of trend curves for each ion representing biosynthetic intermediates from Day 5 to Day 18 for the two marine-derived investigated *Penicillium* fungal strains, MMS460 and MMS388, based on LC-MS data: (**1**) (─) *m/z* 277.0695 at 4.4 min (*r*² = 0.63; 0.55); (**2**) (─) *m/z* 291.0872 at 8.1 min (*r*² = 0.60; 0.07); (**3**) (─) *m/z* 305.0992 at 9.3 min (*r*² = NA; 0.58); (**4**) (─) *m/z* 339.0635 at 10.8 min (*r*² = 0.56; 0.46); (**5**) (─) *m/z* 337.0483 at 9.6 min (*r*² = 0.69; 0.74); (**6**) (─) *m/z* 351.0629 at 10.7 min (*r*² = 0.61; 0.86); (**7**) (─) *m/z* 353.0782 at 10.5 min (*r*² = 0.80; 0.88); analog (**1’**) (─) *m/z* 259.0604 at 10.5 min (*r*² = 0.82; 0.58); analog (**5’**) (─) *m/z* 303.0870 at 9.1 min (*r*² = 0.77; NA); and analog (**7’**) (─) *m/z* 319.1183 at 9.4 min (*r*² = 0.34; 0.80). Correlation coefficients “*r*²” of trend curves are given after each corresponding feature for MMS460 and MMS388, respectively.

Both strains *P. canescens* and *Penicillium* sp. MMS388 produce griseofulvin, the biosynthesis of which has been reported by Cacho *et al*. [51]. Griseofulvin biosynthetic intermediates reported in the literature were searched whithin the whole metabolome of both fungi over time. Ten intermediates, including griseofulvin, were observed. Fitted trend curves of these ten identified compounds were then superimposed on a graphical representation (Figure 5). The first observation was that griseofulvin (**7**) was a type (**d**) metabolite (as previously defined) and its direct precursors mainly appeared as type (**d**) or (**e**) which seemed coherent with its biosynthesis. In the case of *P. canescens* strain, while some intermediates could not be retrieved such as griseophenone C (**3**). Their maximum presence in the crude extracts seemed to follow a general tendency, most of them being delayed in time as they all appeared after 8 to 10 days of growth. The same observation was made in the case of MMS388. However, obvious differences exists between the two strains. First of all, fitted trend curves did not all fit well with the proposed biosynthetic pathway for MMS388, especially for griseophenones B (**4**) and D (**2**). This could be explained by the fact that these two particular compounds are represented by trend curves with the worst fitting (correlation coefficient *r*² < 0.5), because they were detected in small amounts in the extracts and variations among the samples for these particular ions were quite important. Then, it is important to note that for this type of study, more confidence should be placed on highly correlated fitted curves. Secondly, while griseophenone C (**3**) was quite undetectable in *P. canescens*, it was nicely observed in MMS388. On the contrary, desmethyl-dehydro-dechloro-griseofulvin (**5’**) seemed to accumulate in *P. canescens* and was absent in MMS388. This observation led to think that this biosynthetic pathway begins to switch to another one, especially as, at the same time, griseofulvin presence seemed to be reaching an inflection point to later decrease. This highlights that a specific regulation of biosynthetic pathways must exist between strains and shows the interest of time-dependant study for a better understanding of such biosynthetic pathways.

### 2.3. Time-Dependence of Chemical Production

**Figure 6:**
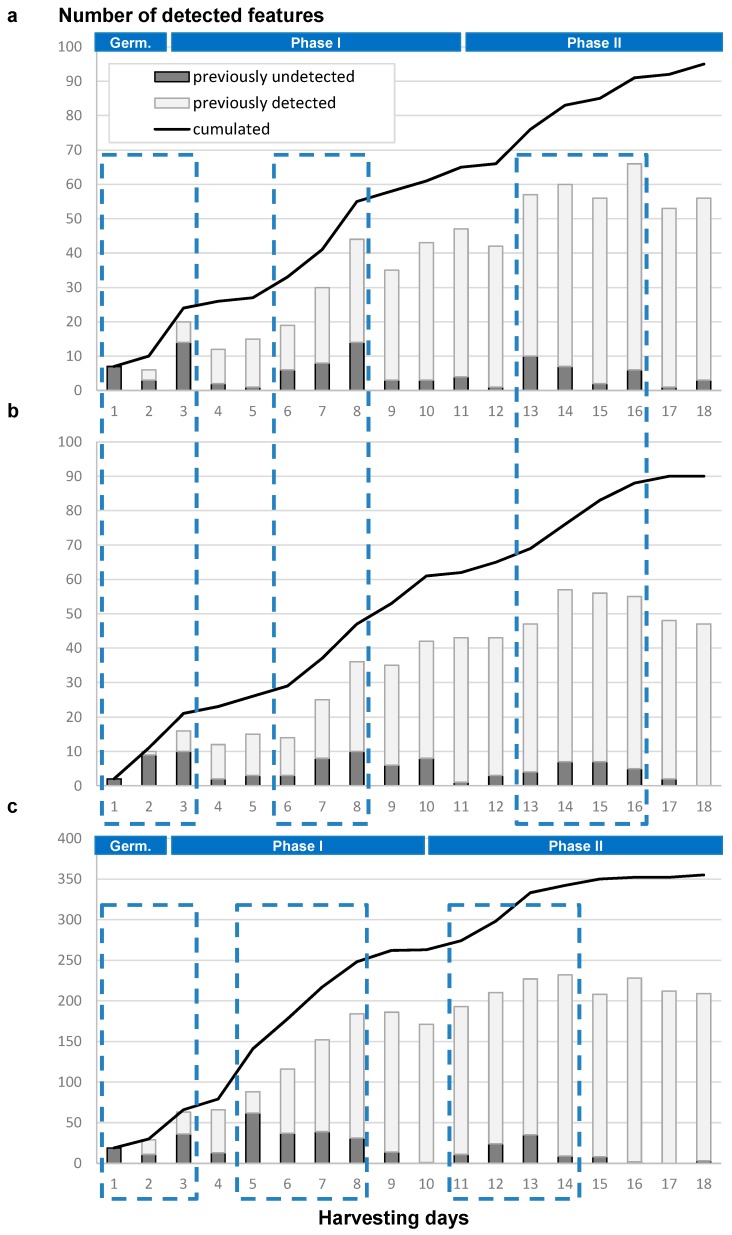
Evolution of the number of detected features in the extracts from Day 1, according to harvesting days using the generated LC-MS peak lists: (**a**) from *P. canescens* MMS460 in the positive mode; (**b**) from *P. canescens* MMS460 in the negative mode; and (**c**) from *Penicillium* sp. MMS388 in the positive mode. The dark grey bars correspond to the number of previously undetected features for each harvesting time, in contrast with light grey bars corresponding to previously detected ones. The black line corresponds to the cumulative number of detected features from Day 1. Periods with maximal number of previously undetected features are highlighted by dotted frames.

As revealed by this study, compound production is highly dependent on growth duration. The optimal harvesting time for catching a maximal chemodiversity is not always easy to anticipate. However, no study has clearly linked harvesting time to chemical potential of strains. To explore this relation, the datasets obtained for *P. canescens* MMS460 and for *Penicillium* sp. MMS388 were analyzed to highlight features undetected in previous days profiles. Over the 18-day period, the total number of detected features (which could be assimilated to compounds) was plotted on a cumulative curve (Figure 6).

This approach led to surprising results in terms of chemical production through time. First of all, the always growing cumulative curve of the total number of detected features suggests that newly produced compounds were detected every day as also highlighted by the dark grey bars in Figure 6. In addition, maximal detection of newly produced features was observed at three stages of the culture for both strains, in the early phase of the fungal growth (Days 1–3), during the mid-phase I (Days 6–8 for MMS460 and Days 5–8 for MMS388), or during the mid-phase II (Days 13–16 for MMS460 and Days 11–14 for MMS388). The plateau phases observed on the cumulative curve between the three previous stages (Figure 6) could correspond to a phase of high production of already observed compounds (light grey bars) and a delay (approximately two days after the transitions observed on the growth curve, Figure 1b) for the induction of new biosynthetic pathways. This shows that potential novelty in terms of secondary metabolites could also be obtained either in early or late growth phases, which correspond to unusual times for classical extraction of fungal material.

In conclusion, an optimum screening procedure to access a larger metabolite chemodiversity for natural drug discovery program would be to perform a series of extractions on a same fungal culture at different times covering the different growth phases.

## 3. Experimental Section

### 3.1. Main General Procedures

Solvents used for extraction were distilled analytical grade solvents (dichloromethane and ethyl acetate). The solvents and reagents used for HPLC-HRESIMS were of Ultra Performance Liquid Chromatography (UPLC) grade (methanol, acetonitrile, water, and formic acid).

### 3.2. Fungal Strains

The fungal material came from the MMS laboratory strain collection, consisting of various samples isolated from the French Atlantic coast near the estuary of the Loire River as described previously [52]. *Penicillium canescens* referenced as MMS460 was initially collected in September 1997 from sediments in Le Croisic (France). *Penicillium* sp. referenced as MMS388 was initially collected in January 1997 from mussels (*Mytilus edulis*) in La Prée (France). After macroscopic and microscopic observations the strains were identified according to a molecular biological protocol by DNA amplification and sequencing of the ITS and β-tubulin regions (GenBank accession number for MMS388: KF953979 and KF953982; MMS460: KU720405 and KU720399). They all have a voucher specimen conserved both at room temperature on agar under sterile paraffin oil and at −20 °C.

### 3.3. Culture Conditions

Cultivation of the strains was performed on seawater semi-solid Yeast Extract Saccharose medium (YES medium) containing 150 g/L saccharose, 20 g/L yeast extract, 0.005 g/L copper sulfate (CuSO_4_·5H_2_O), 0.5 g/L magnesium sulfate (MgSO_4_·7H_2_O), 0.01 g/L zinc sulfate (ZnSO_4_·7H_2_O) and 20 g/L agar. Artificial sea water was reconstituted from the Coral Reef^®^ salts at 33 g/L. Seeding was performed on 90 mm ∅-Petri dishes containing 25 mL of solid culture medium and following 3 points fungal spores deposit in the solid state. Altogether, 90 dishes corresponding to 5 replicates per day of observation were prepared together with 5 replicates of non-seeded dishes corresponding to the controls. The culture was then maintained at 27 °C.

### 3.4. Sample Preparation

Micro-scale extraction was performed for each Petri dish on three 7 mm diameter-plugs obtained on 3 different points on the plate, namely one in the middle of a colony, one on the edge of a colony as far as possible from another colony and one in the vicinity of another colony. To avoid modification on fungal metabolism due to plug sampling, processed plates were not used for further experiments. The crushed gelose and mycelium were extracted twice with 1 mL of a CH_2_Cl_2_/EtOAc (50:50) solvent mixture after 60-min and 30-min ultrasounds treatment respectively. The supernatants were combined and filtered over a 0.2 μm regenerated cellulose membrane to remove spores. Solvents were removed under a nitrogen stream and the crude extracts were freeze-dried before analysis. This process was applied to 5 dishes each day for 18 consecutive days. Media without inoculated fungi were also extracted at different times, namely 5, 10 and 15 days following the same protocol.

### 3.5. HPLC-HRMS Analysis

The crude extracts were analyzed at 1 mg/mL in UPLC grade MeOH, following a 2 μL injection on a UFLC-HRESIMS (IT-TOFMS) Shimadzu instrument (Prominence Ultra Fast Liquid Chromatography coupled to High Resolution Electrospray Ionization Mass Spectrometry combining Ion trap and Time of Flight analyzers). Blank samples with UPLC grade MeOH were also injected randomly during the analyses sequences. Elution was performed on a Kinetex™ C18 column (100 × 2.1 mm, 2.6 μm) maintained at 40 °C and using the following gradient: 15% ACN (+0.1% FA) during 2 min; from 15% ACN (+0.1% FA) to 100% ACN (+0.1% FA) in 23 min; hold at 100% ACN (+0.1% FA) for 5 min; and back to the initial conditions and equilibration for the last 5 min. The detection using ESI was performed both in positive and negative modes with a coupled ion trap and TOFMS analyzer in the mass range *m/z* 100–1000 with a mass accuracy of 7 ppm and a resolution of 10,000 at *m/z* 500. MS^2^ acquisition was added in the protocol in the positive mode for a selection of samples to improve compounds identification. In the case of MMS388 samples were analyzed at random along with QC samples, following LC-MS metabolomics guidelines [53].

### 3.6. Data Treatment

Automatic feature detection was performed between 1 and 30 min with MZmine 2 software [45] using the parameters selected according to the TOFMS detector. Peaks with a width of at least 0.03 min and an intensity greater than 10,000 counts (both in negative and positive modes) were picked with a 10 ppm *m/z* tolerance and the generated peak lists were deconvoluted. Deisotope filtering was applied using the “isotopic peaks grouper” module with tolerance parameters adjusted to 0.3 min and 10 ppm. Feature alignment were achieved with the “Join Aligner” module at a *m/z* tolerance of 10 ppm and a retention time tolerance of 0.4 min followed by gap filling using “Gapfiller” module, yielding a combined dataset. The features detected from blank samples and non-inoculated agar samples were removed from the generated matrix to focus on the features really corresponding to the fungus production. Matrices sizes were 108 × 54 (detected features × samples) and 108 × 54 for MMS460 in positive and negative ionization, respectively; and 423 × 54 and 23 × 54 for MMS388 in positive and negative ionization, respectively. Prior to data analysis, data were normalized according to the total amount of extract to avoid the amplification of compound presence due to a low extract amount at early growth stages. SIMCAP software was used for multivariate data analysis (PCA with UV scaling, see Supplementary Material for PCA parameters) as well as Metaboanalyst 3.0 [54] and more particularly their time series data analysis module [37] to create heatmaps with hierarchical clustering of the features with regards to the days of extraction.

### 3.7. Compounds Identification

Griseofulvin (**7**) was isolated from *P. canescens* MMS460 and a standard was purchased from Sigma-Aldrich. This allowed comparison of NMR, retention time, UV and mass spectra and identification of this compound without a doubt in LC-MS traces (level 1 according to Creek *et al.* [55]). Amauromine (*m/z* 509.2907 at 16.75 min) was also isolated from *P. canescens* MMS460. NMR and MS data were in accordance with the literature [56,57] and allowed the identification of this compound in the profiles (level 1 according to Creek *et al.* [55]). Norlichexanthone (**1’**) was isolated from another strain of *Penicillium* sp. MMS351 in the laboratory and identified after NMR and MS experiments, which were in accordance with the literature [58,59] (level 1 according to Creek *et al.* [55]). Other compounds were annotated based on MS, MS^2^ data, isotopic patterns and UV spectra obtained through the DAD (level 2–4 according to Creek *et al.* [55], see Supplementary Materials).

## 4. Conclusions

In the present study, two *Penicillium* strains were cultivated over an 18 days period. Every day fungal material with culture media was extracted and profiled by HPLC-HRMS. This experiment revealed that time is an important factor to take into account for natural product production by fungi. Important metabolite production differences were observed through time. When epigenetic modifiers, co-cultures or OSMAC approaches have been more and more studied, time-scale metabolomics remains an under-explored but promising field to investigate (alone or in combination) to access fungal natural products in a larger chemical space.

These studies revealed that time metabolomic studies could help in the description of natural products biosynthesis, by studying the occurrence and the delay in apparition of intermediates over time. Whenever a biosynthetic route is well described, regulations can be quite different from one organism to another. To access a higher diversity in the purification of the different analogs or biosynthetic intermediates from a series, increasing the number of strains to be studied would be valuable.

In addition, the growth time parameter is a key parameter to access undetected chemical entities. Both early and late growth time are usually unexplored culture conditions. Therefore this important parameter should be taken into consideration in bioprospecting studies to further extend the natural products chemical space produced by microorganisms.

Exploring unusual growth duration should lead to improved chemical novelty detection in bioprospection process. Multiplying sampling during a fungal culture should balance the fact that at a given time only subsets of biosynthetic pathways encoding for secondary metabolite production are ever expressed.

## Supplementary Materials

The following are available online at www.mdpi.com/1660-3397/14/5/103/s1, Table S1: Peak annotation from LC-UV-HRMS/MS data, Table S2: Simca parameters for PCA scores plot, Figure S1: Isolated Amauromine NMR HSQC spectrum (in CDCl_3_, 500, 125 MHz), Figure S2: Isolated Amauromine UV spectrum (from DAD in H_2_O/ACN 0.1% formic acid), Figure S3: Isolated Amauromine MS spectra, Figure S4: Isolated Desmethyl-dehydro-griseofulvine A NMR HSQC spectrum (in MeOD, 500, 125 MHz), Figure S5: Isolated Desmethyl-dehydro-griseofulvine A UV spectrum (from DAD in H_2_O/ACN 0.1% formic acid), Figure S6: Isolated Desmethyl-dehydro-griseofulvine A MS spectra, Figure S7: Isolated Norlichexanthone ^1^H NMR spectrum (in MeOD, 500 MHz), Figure S8: Isolated Norlichexanthone ^13^C NMR spectrum (in MeOD, 125 MHz), Figure S9: Isolated Norlichexanthone UV spectrum (from DAD in H_2_O/ACN 0.1% formic acid), Figure S10: Isolated Norlichexanthone MS spectra, Figure S11: Hierarchical clustering of the features observed from day 1 (D1) to day 18 (D18) in a marine-derived *Pemicillium* sp. MMS388 in the positive mode.
