# Supplementary material for: Time Dependency of Chemodiversity and Biosynthetic Pathways: An LC-MS Metabolomic Study of Marine-Sourced Penicillium
Source: Mar Drugs. 2016 May 21;14(5):103. doi: 10.3390/md14050103 (PMC4882577; doi:10.3390/md14050103)
Supplement: Supplementary file 1 [file marinedrugs-14-00103-s001.pdf]

Supplementary Materials: Time Dependency of Chemodiversity and Biosynthetic Pathways: An LC-MS Metabolomic Study of Marine-Sourced *Penicillium*

Catherine Roullier, Samuel Bertrand, Elodie Blanchet, Mathilde Peigné, Thibaut Robiou du Pont, Yann Guitton Yves François Pouchus and Olivier Grovel

Table S1. Peak annotation from LC-UV-HRMS/MS data.
Table S2. Simca parameters for PCA scores plot.
Figure S1. Isolated Amauromine NMR HSQC spectrum (in $CDCl_3$, 500, 125 MHz).
Figure S2. Isolated Amauromine UV spectrum (from DAD in H_2O/ACN 0.1% formic acid).
Figure S3. Isolated Amauromine MS spectra.
Figure S4. Isolated Desmethyl-dehydro-griseofulvine A NMR HSQC spectrum (in MeOD, 500, 125 MHz).
Figure S5. Isolated Desmethyl-dehydro-griseofulvine A UV spectrum (from DAD in H_2O/ACN 0.1% formic acid).
Figure S6. Isolated Desmethyl-dehydro-griseofulvine A MS spectra.
Figure S7. Isolated Norlichexanthone ^1H NMR spectrum (in MeOD, 500 MHz).
Figure S8. Isolated Norlichexanthone ^{13}C NMR spectrum (in MeOD, 125 MHz).
Figure S9. Isolated Norlichexanthone UV spectrum (from DAD in H_2O/ACN 0.1% formic acid).
Figure S10. Isolated Norlichexanthone MS spectra.
Figure S11. Hierarchical clustering of the features observed from day 1 (D1) to day 18 (D18) in a marine-derived *Pemicillium* sp. MMS388 in the positive mode.

Table S1. Peaks annotation from LC-UV-HRMS/MS data.

Rt (min)	m/z	Type of adduct	MS^2 fragments	Molecular Formula	UV data λ_{max} in nm (from DAD)	Identification	MSI level of confidence	Standard	Observed in Strains
4.4	277.0695	$[M+H]^+$	235.0383	$C_{14}H_{12}O_5$	200 (100), 220 (sh), 269 (40), 302 (sh)	2,2',4,4',6-pentahydroxy-6'-methylbenzophenone (**1**)	2	no	MMS460 & MMS388
8.1	291.0872	$[M+H]^+$	167.0373	$C_{15}H_{14}O_6$	204 (100), 295 (27)	griseophenone D (**2**)	2	no	MMS460 & MMS388
8.7	506.2543	$[M+H]^+$	173.0636, 202.0532, 254.1922, 279.1461, 319.1654, 339.1907, 367.1857	$C_{30}H_{35}NO_6$	206 (100), 251 (72), 325 (38)	decaturin A, decaturin B	4	no	MMS460
9.1	303.0870	$[M+H]^+$	275.0815, 285.0742	$C_{16}H_{14}O_6$	206 (100), 292 (15), 342 (20)	desmethyl-dehydro-dechloro-griseofulvin (**5'**)	2	no	MMS460 & MMS388
9.3	305.0992	$[M+H]^+$	165.0531	$C_{16}H_{16}O_6$	206 (100), 291 (21)	griseophenone C (**3**)	2	no	MMS388
9.4	319.1183	$[M+H]^+$	181.0498, 251.0916, 287.1124	$C_{17}H_{18}O_6$	205 (100), 293 (25)	dechloro-griseofulvin (**7'**)	2	no	MMS460 & MMS388
9.6	337.0483	$[M+H]^+$	294.0293, 306.0296, 322.0241	$C_{16}H_{13}ClO_6$	213 (90), 291 (100), 340 (sh)	desmethyl-dehydro-griseofulvin A (**5**)	1	yes - isolated	MMS460 & MMS388
9.6	504.2386	$[M+H]^+$	202.0506, 213.0881, 250.1093, 267.1374, 321.1687, 347.1611, 365.1688, 383.1937	$C_{30}H_{33}NO_6$	205 (100), 269 (15), 330 (15)	15-deoxyoxalicine B	3	no	MMS460
9.7	403.1037	$[M+H]^+$	195.0656, 209.0450, 371.0699, 385.0936	$C_{20}H_{18}O_9$	207 (100), 315 (40)	acetylversiconol, variecolorquinone, integrastatin B	4	no	MMS460
10.5	259.0604	$[M+H]^+$	191.0703, 217.0416, 244.0366	$C_{14}H_{10}O_5$	202 (75), 241 (100), 267 (sh), 312 (57), 347 (sh)	norlichexanthone (**1'**)	1	yes - isolated	MMS460 & MMS388
10.5	353.0780	$[M+H]^+$	165.0569, 215.0111, 285.0546, 321.0509	$C_{17}H_{17}ClO_6$	211 (100), 236 (77), 293 (86), 338 (sh)	griseofulvin (**7**)	1	yes - purchased	MMS460 & MMS388
10.7	351.0629	$[M+H]^+$	291.0485, 320.9987, 336.0378	$C_{17}H_{15}ClO_6$	203(100), 295 (30)	dehydro-griseofulvin (**6**)	2	no	MMS460 & MMS388
10.8	339.0635	$[M+H]^+$	165.0583, 336.4453	$C_{16}H_{15}ClO_6$	203(100), 295 (30)	griseophenone B (**4**)	2	no	MMS460 & MMS388
11.3	490.2599	$[M+H]^+$	202.0576, 321.1883, 339.1960, 369.2077	$C_{30}H_{35}NO_5$	207 (100), 250 (sh), 280 (sh), 314 (10)	decaturin C, decaturin G	3	no	MMS460
12.5	488.2437	$[M+H]^+$	173.0688, 202.0460, 253.1194, 307.1691, 349.1821, 367.1879	$C_{30}H_{33}NO_5$	207 (100), 249 (8), 285 (7), 334 (7)	15-deoxyoxalicine A	3	no	MMS460
13.5	273.0763	$[M+H]^+$	213.0557, 230.0594, 241.0484, 258.0554	$C_{15}H_{12}O_5$	207 (100), 239 (60), 310 (34)	dihydroxy-methoxy-methylxanthone	3	no	MMS460

Table S1. *Cont.*

Rt (min)	m/z	Type of adduct	MS^2 fragments	Molecular Formula	UV data λ_{max} in nm (from DAD)	Identification	MSI level of confidence	Standard	Observed in Strains
14.0	478.2953	$[M+H]^+$	202.0492, 259.2392, 271.2450, 339.2356, 460.2848	$C_{30}H_{39}NO_4$	208 (100), 258 (20), 286 (10), 329 (8)	predecaturin E	3	no	MMS460
14.1	515.2635	$[M+H]^+$	487.2651	$C_{29}H_{38}O_8$	210 (sh), 288	citreohybridone B - isocitreohybridone B	3	no	MMS460
14.8	474.2646	$[M+H]^+$	202.0492, 255.2078, 353.2101	$C_{30}H_{35}NO_4$	210 (100), 255 (sh), 285 (sh), 334 (sh)	decaturin D	3	no	MMS460
14.9	476.2798	$[M+H]^+$	202.0497, 257.2249	$C_{30}H_{37}NO_4$	205 (100), 256 (sh), 287 (sh), 328 (sh)	decaturin E	3	no	MMS460
15.4	507.229	$[M+H]^+$	nd	$C_{32}H_{30}N_2O_4$	nd	too many (asterriquinone, cochliodinol, isocochliodinol, neocochliodinol, hinnuliquinone …)	4	no	MMS460
16.3	600.3319	$[M+H]^+$	496.2460, 514.2559	$C_{37}H_{45}NO_6$	259 (100), 290 (sh)	penitremone A	3	no	MMS460
16.8	509.2907	$[M+H]^+$	198.1282, 254.0912, 266.1906, 373.1656, 385.1656, 441.2272, 453.2236	$C_{32}H_{36}N_4O_2$	205 (100), 244 (20), 297 (10)	amauromine	1	yes - isolated	MMS460
19.1	584.3380	$[M+H]^+$	496.2465, 514.2569, 548.3193, 566.3250	$C_{37}H_{45}NO_5$	211 (100), 287 (24)	penitremone C	3	no	MMS460

nd: not determined (usually because of co-elution or low signal)

Table S2. Simca parameters for PCA scores plot.

	Scaling	Components	R2X(cum)	Eigenvalue
MMS460	UV	7	0,823	1,43
	Pareto	4	0,867	1,69
MMS388	UV	6	0,733	1,48
	Pareto	5	0,847	1,42
D5 excluded	UV	5	0,64	1,79
D5 excluded	Pareto	4	0,82	1,58

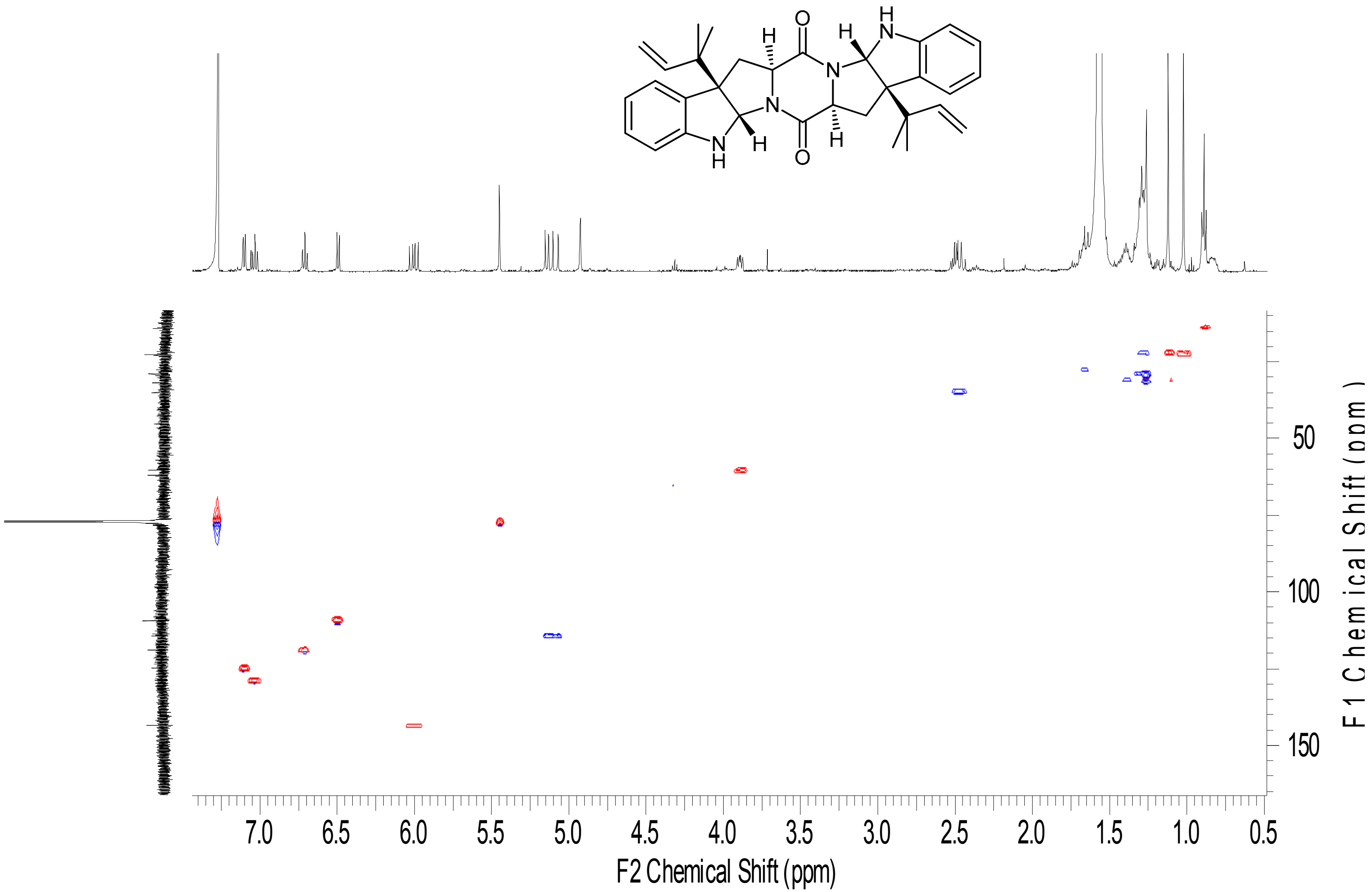

Figure S1. Isolated Amauromine NMR HSQC spectrum (in $CDCl_3$, 500, 125 MHz).

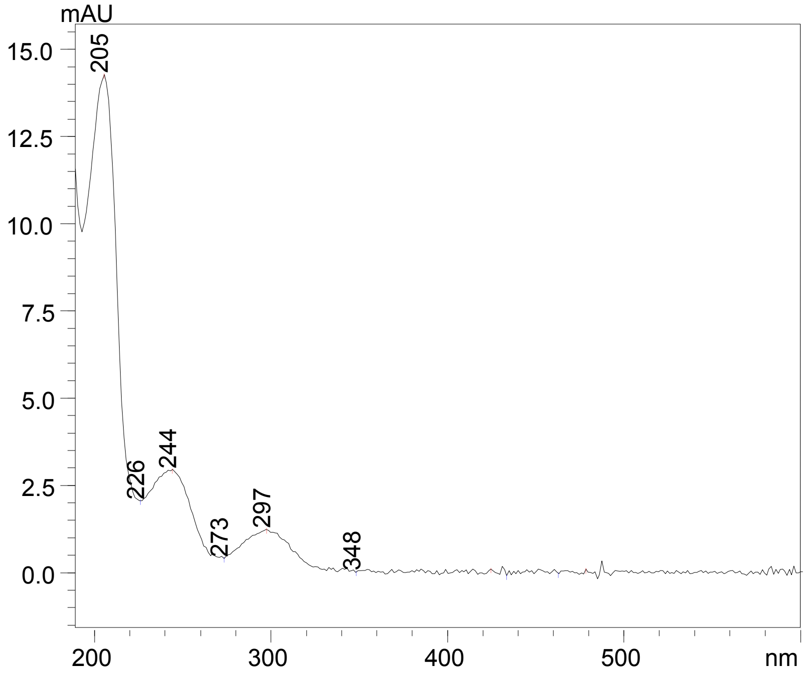

Figure S2. Isolated Amauromine UV spectrum (from DAD in H_2O/ACN 0.1% formic acid).

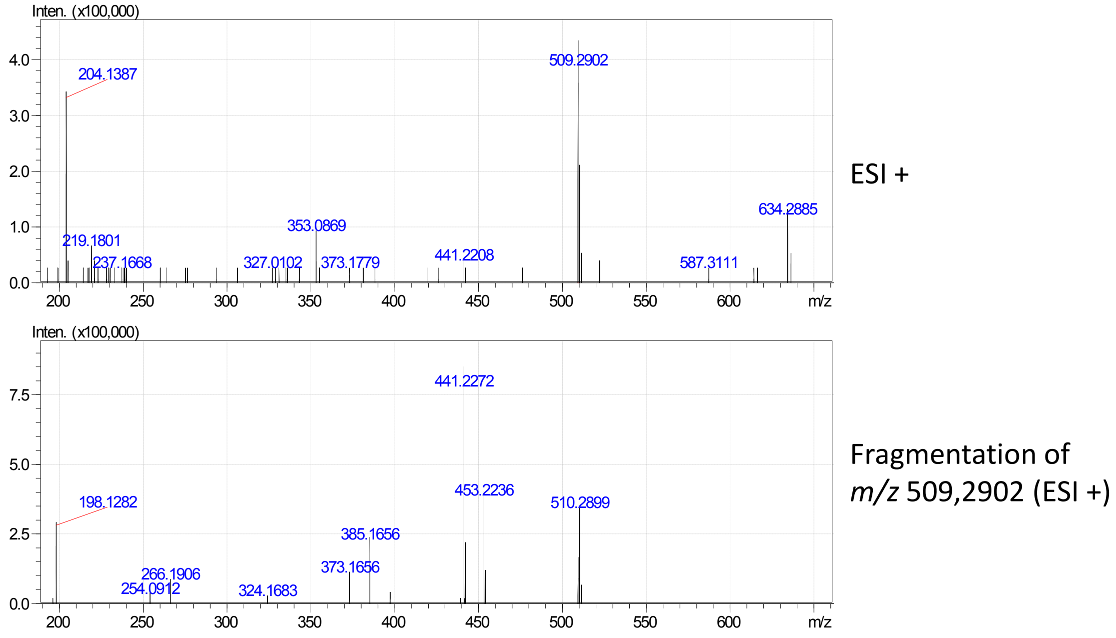

Figure S3. Isolated Amauromine MS spectra.

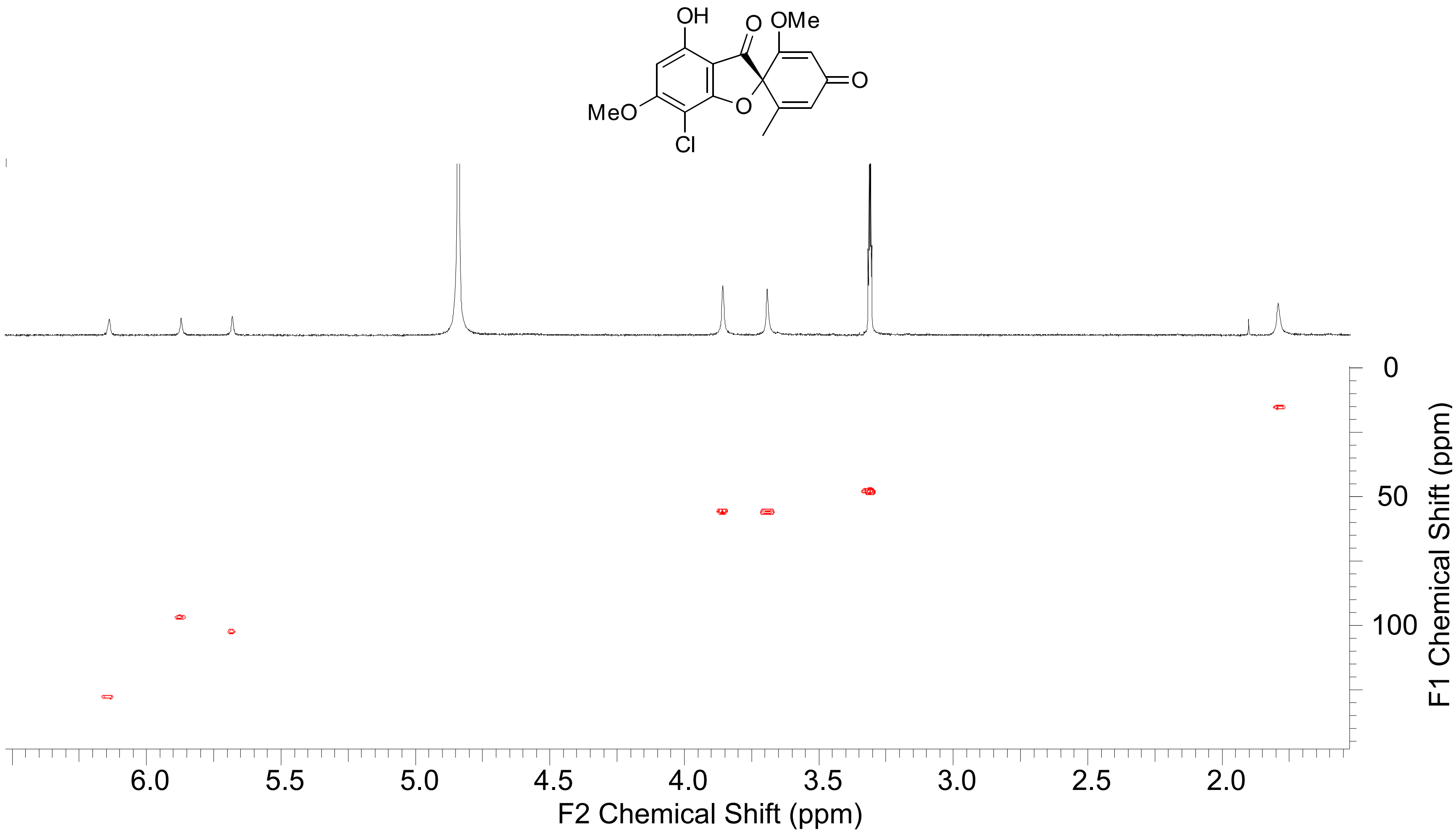

Figure S4. Isolated Desmethyl-dehydro-griseofulvine A NMR HSQC spectrum (in MeOD, 500, 125 MHz).

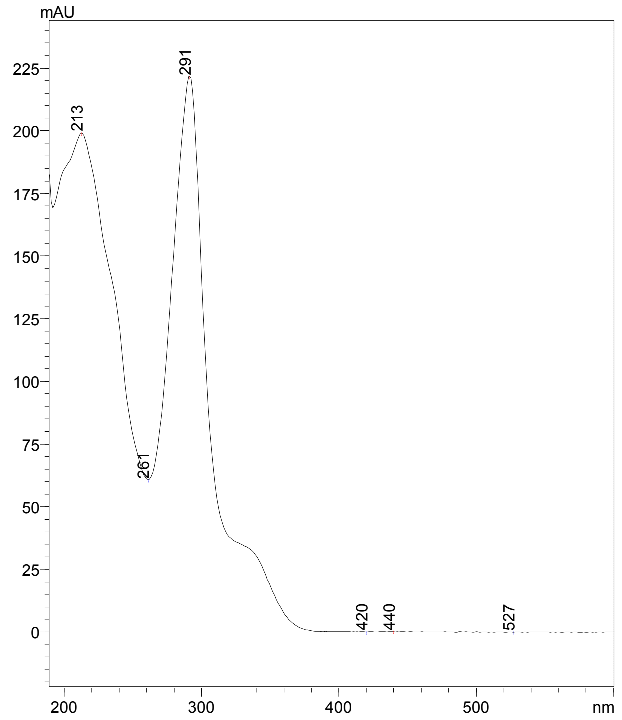

Figure S5. Isolated Desmethyl-dehydro-griseofulvine A UV spectrum (from DAD in H_2O/ACN 0.1% formic acid).

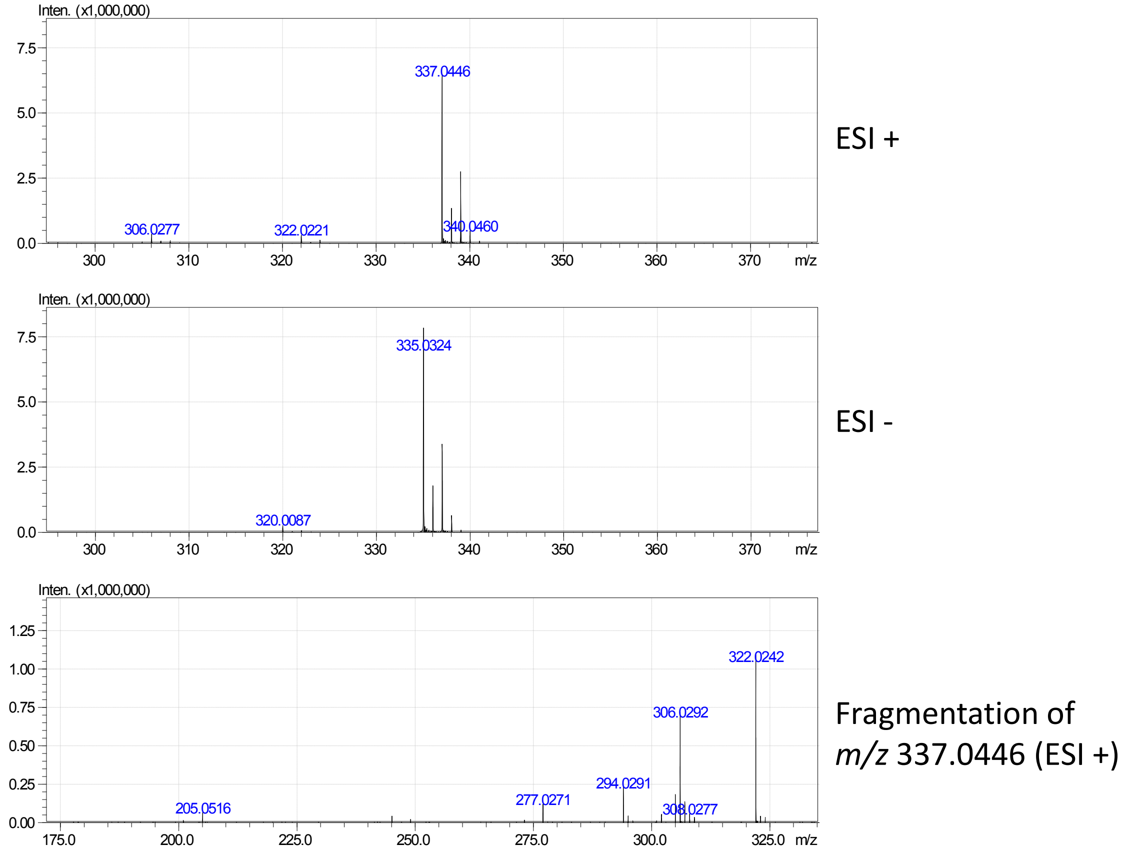

Figure S6. Isolated Desmethyl-dehydro-griseofulvine A MS spectra.

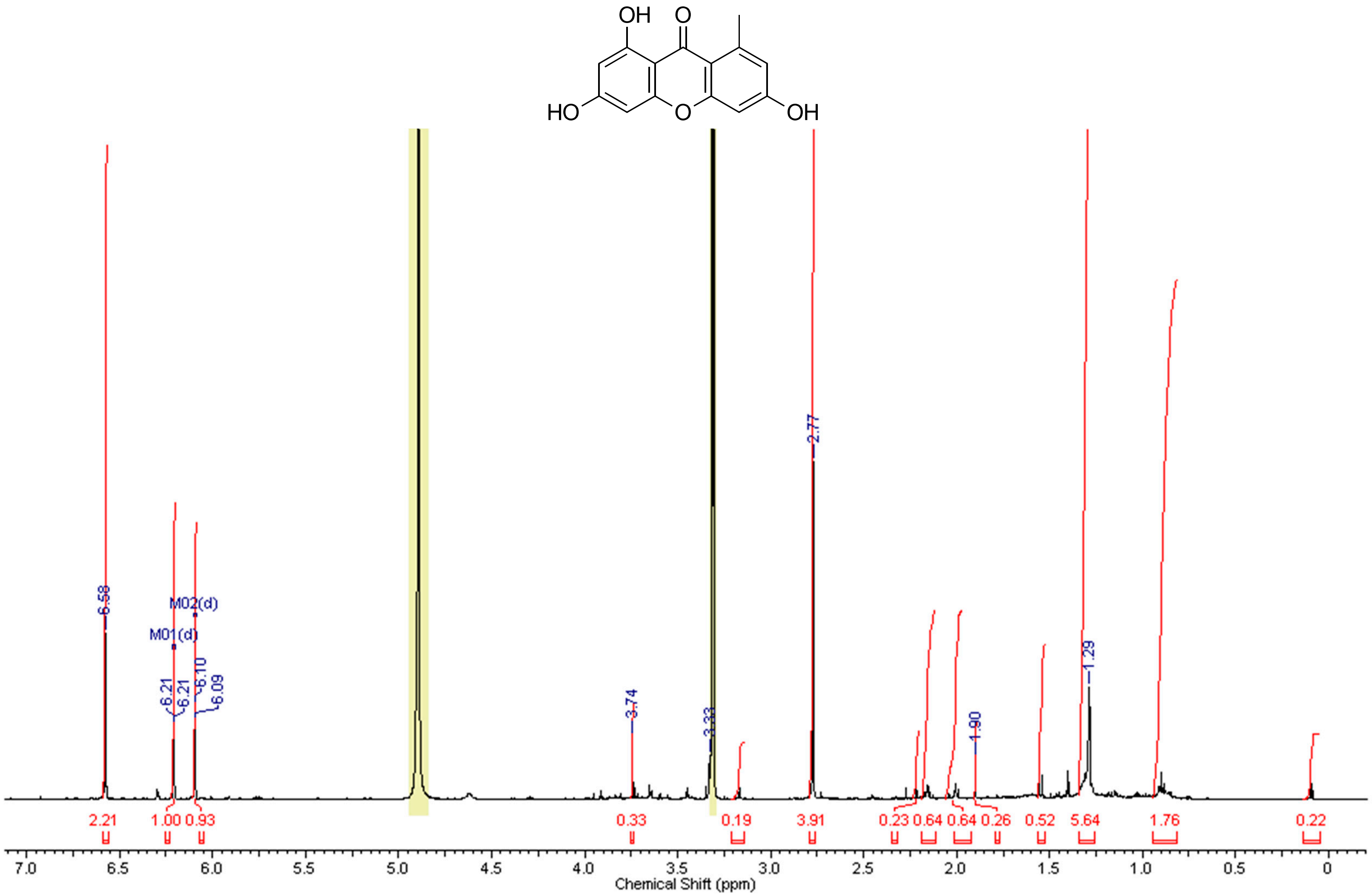

Figure S7. Isolated Norlichexanthone ^{1}H NMR spectrum (in MeOD, 500 MHz).

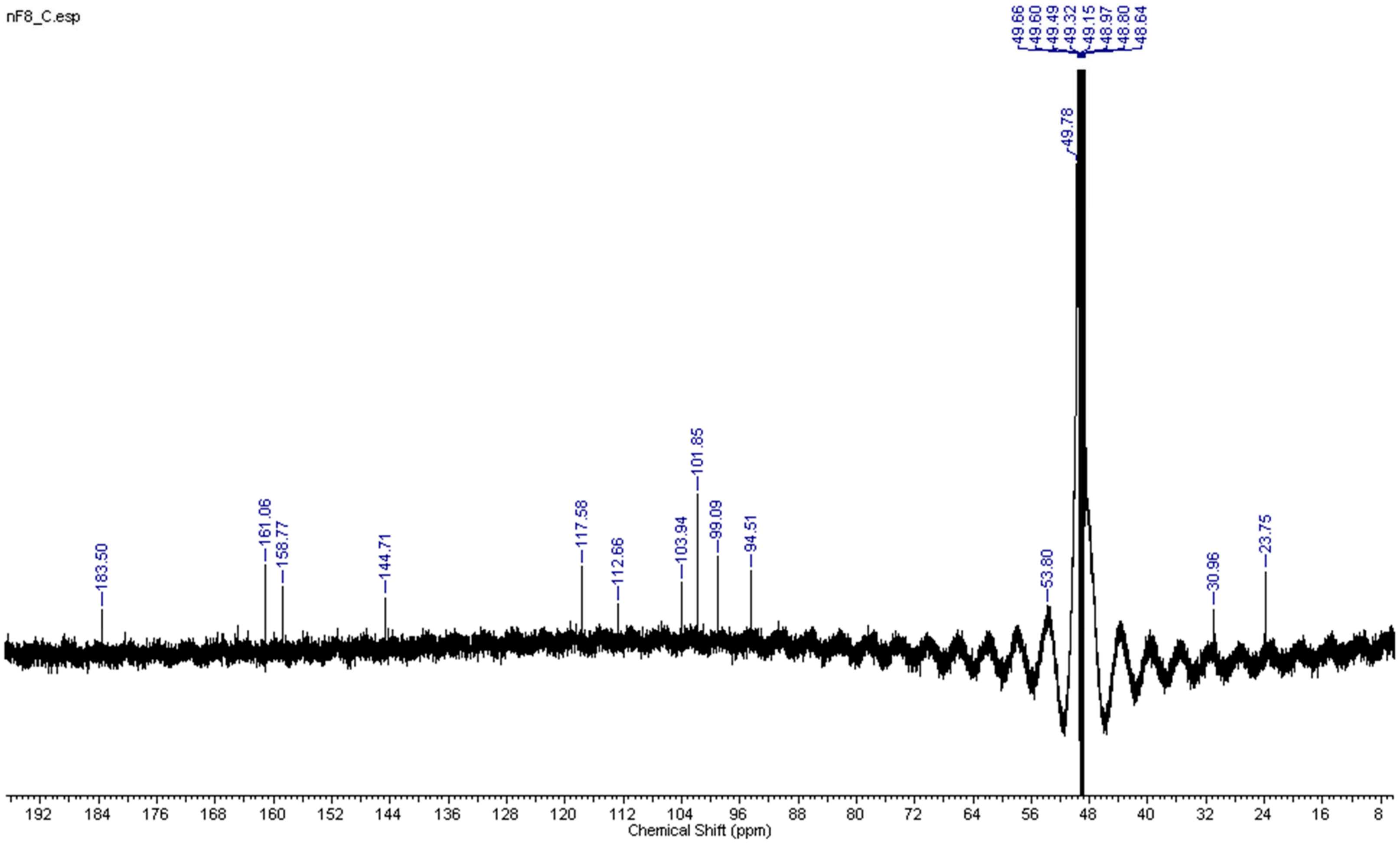

Figure S8. Isolated Norlichexanthone ^{13}C NMR spectrum (in MeOD, 125 MHz).

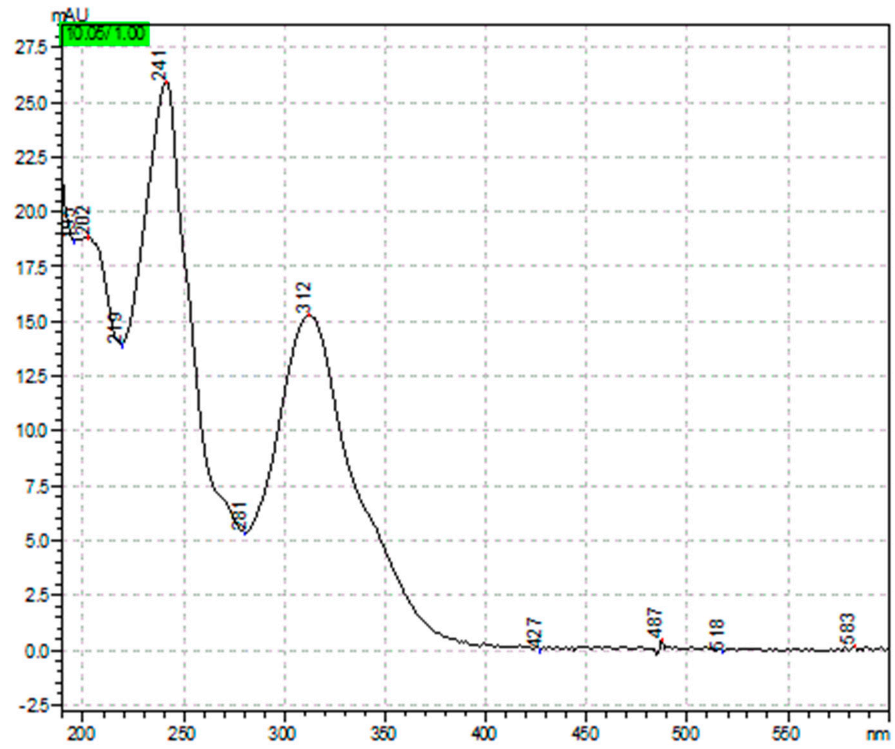

Figure S9. Isolated Norlichexanthone UV spectrum (from DAD in H_2O/ACN 0.1% formic acid).

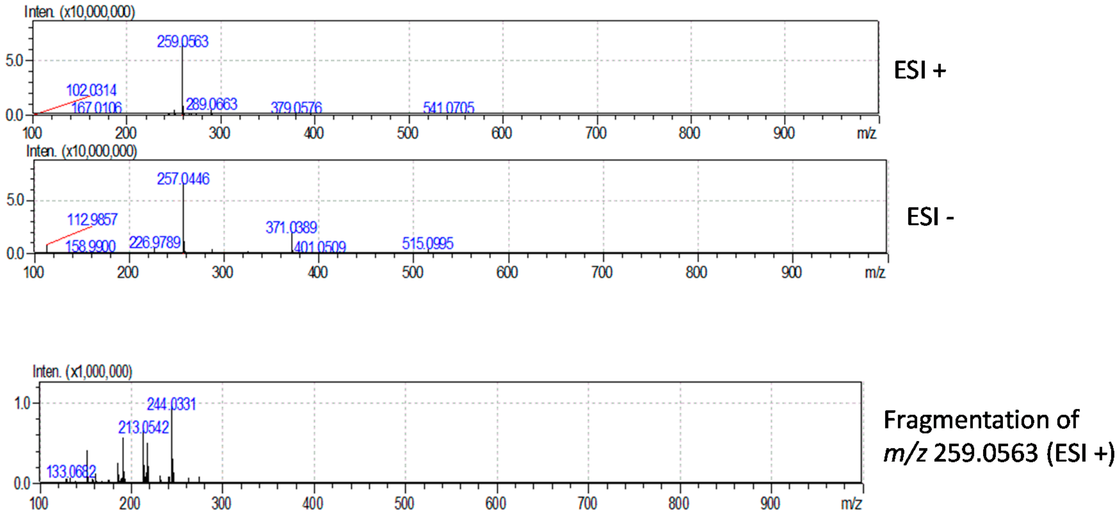

Figure S10. Isolated Norlichexanthone MS spectra.

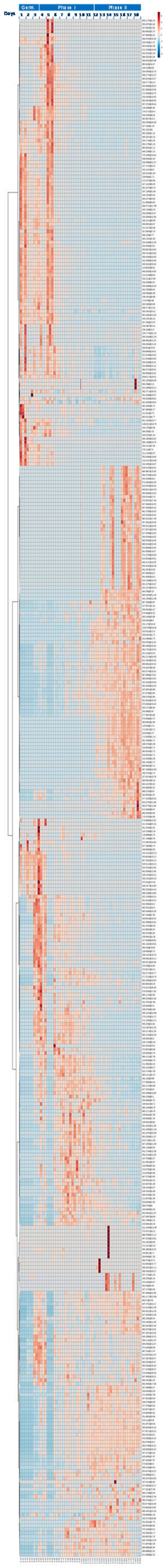

Figure S11. Hierarchical clustering of the features observed from day 1 (D1) to day 18 (D18) in a marine-derived *Pemicillium* sp. MMS388 in the positive mode.
